# Potential prognostic biomarkers in restenosis of peripheral arteries identified through comprehensive ceRNA network analysis

**DOI:** 10.3389/fgene.2025.1597644

**Published:** 2025-12-01

**Authors:** Yulin Miao, Zongxin Li, Qianqian Sun, Gang Zhao, Guohang Shen, Haobo Zhang, Fengli Gao

**Affiliations:** 1 Department of Vascular Surgery, General Hospital of Ningxia Medical University, Yinchuan, Ningxia, China; 2 The First Clinical Medical College, Ningxia Medical University, Yinchuan, Ningxia, China

**Keywords:** peripheral arterial disease, restenosis, lncRNA, ceRNA network, prognostic biomarkers

## Abstract

Peripheral artery disease (PAD) is one of the leading causes of amputation and mortality worldwide, with restenosis being a common complication following vascular interventional therapy that significantly impacts patient prognosis. This study aims to identify potential biomarkers with clinical and prognostic significance for restenosis of peripheral arteries (RPA). To achieve this, RNA expression levels in stenotic vascular tissues from healthy controls and patients with RPA were integrated and analyzed, identifying a growing number of differentially expressed RNAs for the construction of a competitive endogenous RNA (ceRNA) network for prognostic analysis. The analysis revealed that the RNA expression patterns of samples from patients with RPA were significantly different compared with those of healthy controls. Consequently, the resulting differentially expressed RNAs were used to construct a ceRNA network. Through bioinformatics analysis, a complex regulatory network composed of multiple ceRNA axes was established. The findings indicate that key mRNAs in the network play critical roles in various biological functions. Given the prognostic significance of these six differentially expressed genes in RPA, real-time quantitative fluorescence PCR (qRT-PCR) analysis was performed. These results suggest that these candidate key differentially expressed genes are potential prognostic biomarkers for RPA, and their corresponding mRNAs may serve as potential therapeutic targets.

## Introduction

Peripheral artery disease (PAD) affects approximately 15%–20% of individuals over the age of 70, with restenosis (RS) being a common complication following endovascular treatment for PAD (Jia et al., 2022). Currently, percutaneous transluminal angioplasty (PTA) is a widely used treatment strategy for vascular lesions, due to its minimally invasive nature and effectiveness. However, up to 70% of patients experience restenosis within 1 year, limiting its long-term efficacy. Despite attempts to pharmacologically prevent or reduce RS using drug-coated balloons, the restenosis rate remains high, with nearly 23% of patients developing restenosis within 12 months. RS has become a major obstacle to the long-term success of lower extremity arteries after PTA (Zhou et al., 2021). Therefore, it is of great significance to explore the potential molecular mechanisms of RS development. The identification of prognostic biomarkers for RPA could enhance our understanding of its pathogenesis, and potentially lead to the discovery of novel therapeutic targets.

Long non-coding RNAs (lncRNAs) have been reported to play crucial roles in cardiovascular physiology and pathophysiology, including cardiac development and heart failure (Uchida and Dimmeler, 2015; Bai et al., 2019). Increasing evidence suggests that lncRNAs can interact with other RNA transcripts through the ceRNA regulatory mechanism, acting as molecular sponges for miRNAs. By sharing miRNA response elements (MREs), lncRNAs influence miRNA regulation of target genes and downstream molecular processes (Salmena et al., 2011; Sun et al., 2020). Given the significant role of ceRNAs in various human diseases, exploring ceRNA networks in PAD may provide novel insights into the biological mechanisms of this disease.

In this study, whole transcriptome data of 5 healthy control tissue samples and 5 RPA tissue samples were analyzed to investigate the potential molecular regulatory mechanisms underlying RPA. First, quality control was performed on the self-test data to detect the quality of data sequencing. Then, the differentially expressed genes (DEGs) and differentially expressed non-coding RNAs (DE-ncRNAs) between the RPA group and the Control group were screened in the self-test data to construct a differential regulatory network. Key genes were subsequently identified using multiple Cytoscape algorithms, followed by functional analysis and diagnostic capability evaluation. Furthermore, the relationship between key genes and immune characteristics was examined, and upstream regulatory networks of the key genes were explored through differentially expressed non-coding RNAs to establish a theoretical foundation for the study of key genes. Finally, the expression of key genes was further determined via qRT-PCR experiments, and their corresponding mRNAs may serve as potential therapeutic targets.

## Materials and methods

### Acquisition of transcriptome and clinical sample data of stenotic tissue samples

Transcriptome sequencing was performed on 5 healthy control tissue samples and 5 RPA tissue samples collected for patients after PTA. After filtering, alignment, and merging of the raw sequencing data, the expression matrix of the whole transcriptome for each sample was obtained. The expression matrix is presented in count values, as detailed in the Supplementary File S1. *RawData.xlsx*.

### Quality control and preprocessing of transcriptome sequencing data

Quality control of the raw sequencing data for mRNAs and lncRNAs was conducted using FastQC software (version 0.11.9). Low-quality data, contaminations, and adapter sequences were filtered out. The cleaned sequencing data were then aligned to the reference genome (GRCh38). After the single cell sequencing data was filtered through quality control, the quality of each base in the retained sequencing reads reached the QC30 standard, indicating a base error rate of less than 1/1000. The alignment rates of all sequencing samples were above 95%, suggesting high alignment quality suitable for subsequent transcriptome analysis (Xie et al., 2022; Zhang et al., 2021; Liu et al., 2022). The raw sequencing data processing pipeline of miRNAs was similar to that of mRNAs and lncRNAs, and a total of 2,656 miRNAs were identified across the 10 samples. Detailed information is provided in the Supplementary File S1. *RawData.xlsx*.

### Identification of lncRNA

StringTie software was used to reconstruct transcripts in each sample based on a probability model. The reconstructed transcripts were then compared with the reference transcripts using Cuffcompare software to identify known long-chain lncRNAs and transcripts similar to other non-coding RNAs (ncRNAs) and mRNAs. Transcripts meeting lncRNA-specific characteristics were retained. Candidate lncRNAs were screened based on the criteria of length >200 bp and number of exons ≥2. In addition, the coding potential calculator (CPC) analysis (Kong et al., 2007), coding-non-coding index (CNCI) analysis (Sun et al., 2013), Pfam protein domain analysis (El-Gebali et al., 2019), and PLEK analysis (Li et al., 2014) were used to predict the coding ability of candidate lncRNAs.

### Identification of differentially expressed genes and functional enrichment analysis

To identify DEGs between the Control and RPA groups, differential expression analysis was performed using the edgeR R package (version 3.36.0). The screening criteria for DEGs were P < 0.05 and |Log2FC| ≥2, and differentially expressed lncRNAs (differentially expressed-lncRNAs, DE-lncRNAs), miRNAs (differentially expressed-miRNAs, DE-miRNAs), and mRNAs (differentially expressed-mRNAs, DE-mRNAs) were finally obtained. Then, the ggplot2 R package (version 3.3.5) was used to generate volcano plots illustrating gene expression patterns, and the pheatmap R package (version 1.0.12) was employed to create heatmaps of differentially expressed genes.

### Functional enrichment analysis of DEGs

Functional enrichment analysis on DE-lncRNAs and DE-mRNAs was performed using the clusterProfiler R package (version 4.0.2), including Gene ontology (GO) and Kyoto Encyclopedia of Genes and Genomes (KEGG) analysis. The GO enrichment analysis mainly describes biological processes (BP), cellular components (CC), and molecular functions (MF) associated with genes, while the KEGG pathway analysis was used to identify the biological pathways associated with genes. The miEAA database (https://ccb-compute2.cs.uni-saarland.de/mieaa/) is an online tool for miRNA enrichment analysis supporting 10 species, including humans, to explore the biological significance represented by each miRNA (Aparicio-Puerta et al., 2023). The LncSEA database (https://bio.liclab.net/LncSEAv2/index.php) is a resource focusing on human ncRNAs and offers direct annotation and enrichment analysis of submitted lncRNAs. In this study, p <0.05 and count value >1 were used as screening thresholds to obtain GO and KEGG enrichment results of DEGs.

### Construction of ceRNA network

The tarbase database (https://dianalab.e-ce.uth.gr/tarbasev9) was used to predict the downstream miRNAs of DE-lncRNAs, this database contains potential interactions between lncRNAs and miRNAs (Vlachos et al., 2015). The target mRNAs associated with these miRNAs were predicted based on the miRWalk database (http://mirwalk.umm.uni-heidelberg.de/) (Elboim-Gabyzon et al., 2022). The predicted miRNAs and DE-miRNAs, as well as the predicted mRNAs and DE-mRNAs, were intersected to form lncRNAs-miRNAs and miRNA-mRNA relationship networks, respectively. The lncRNAs and mRNAs in these two networks were selected, and the correlation between the two was analyzed by spearman correlation analysis (Kohl et al., 2011). The correlation pairs with an absolute value of the correlation coefficient greater than 0.6 were used to form the lncRNAs-mRNAs relationship network. The Cytoscape software (version 3.7.2) was used to construct the final ceRNA network by combining the above three correlation pairs (Shen et al., 2014).

### Identification and evaluation of key genes

The STRING database (https://string-db.org) was used to construct the PPI network of differentially expressed mRNAs. The MCODE plugin of Cytoscape was further used to screen genes in the key regulatory network based on five algorithms (MNC, DMNC, EPC, BottleNeck, Betweenness) (Alam et al., 2022; Jiang and Han, 2024; Pakkir Shah et al., 2025; Qian et al., 2024). The top 10 genes screened by each algorithm were intersected to form a set of key genes. The ClueGO plugin of Cytoscape was used to perform functional enrichment analysis on the key genes (Li et al., 2020).

### Gene set enrichment analysis (GSEA)

In order to further explore some related signal pathways and potential biological mechanisms of key genes, clusterProfiler R package (version 4.0.2) and org.Hs.eg.db R package (version 3.13.0) were used to perform single gene GO and KEGG enrichment analysis on key genes respectively. The GSEA enrichment gene set file was downloaded from the GSEA official website (http://www.gsea-msigdb.org/gsea/msigdb) (Peng et al., 2022; Cao and Kagan, 2023), specifically GO: c5.go.v7.4.entrez.gmt and KEGG: c2.cp.kegg.v7.4.entrez.gmt. The samples were divided into high and low expression groups based on the median expression value of key genes, and then GSEA enrichment analysis was conducted on all genes in both groups, with the threshold of absolute value NES>1, NOM P <0.05, and q <0.25.

### Immune infiltration analysis

Single sample GSEA (ssGSEA) is a method proposed mainly for single samples that cannot be analyzed by GSEA. The principle is similar to GSEA gene enrichment analysis. Through the ssGSEA method, the immune cells or immune functions of each sample, and the activity of immune pathways were obtained, and the samples were grouped based on their immune activity. Based on 24 immune cell sets, the ssGSEA algorithm is used to calculate the abundance percentage of infiltrating immune cells in each sample. Then, the proportion of infiltrating immune cells in the sample was visualized based on the calculation results.

### Construction of key gene-drug interaction network

In order to explore potential therapeutic drugs for key gene-related diseases, targeted drugs for proteins encoded by key genes were identified through the TissueNexus database, and a gene-drug interaction network was constructed.

### RNA extraction and validation of key genes by real-time quantitative fluorescence PCR (qRT-PCR)

In this study, tissue samples from 10 cases of rapid and slow RPA after PTA surgery were obtained from patients. Total RNA was extracted from the stenotic tissue samples using the RNeasy kit (Qiagen 74104). Subsequently, reverse transcription was performed using a reverse transcription kit (AG11706, AG, China) following the instructions. cDNA was amplified using the SYBR premixed Ex Taq kit (AG11718, AG, China), and GAPDH was used as an internal reference gene to calculate the RNA expression levels of key genes. All primer sequences are shown in Table 1.

**TABLE 1:** Gene primer sequences.

Primers	Sequences
KCNC1 F	AGA​TAC​GGA​CCC​TGC​TTC​CT
KCNC1 R	TAC​TCT​GTC​CGA​GGG​TGA​GG
FRMPD4 F	GGC​TGC​ATA​TTG​AAT​CCA​CAG​GAC
FRMPD4 R	CAT​CTC​CAC​CTT​CCG​AGG​AG
ATP2B2 F	AAC​CGT​GCA​GGA​TTG​GTG​G
ATP2B2 R	TGA​GCT​CCA​AGA​GGT​GTC​CT
NOS1 F	AGC​AAA​GCA​ACT​GCC​AAG​GA
NOSI R	TCA​GTG​CAT​CCC​GTT​TCC​AA
SHC3 F	TGT​GCT​CGT​GAT​GTC​ATG​CT
SHC3 R	GGC​TTG​TCA​CAC​TCC​AAC​AC
FAIM2 F	GGA​TGT​TGC​TAG​AAC​CCA​CG
FAIM2 R	CTT​CTT​CTC​GCC​ATG​CAC​CT
Internal reference-Gapdh F	GAC​CCC​TTC​ATT​GAC​CTC​AAC
Internal reference-Gapdh R	GCC​ATC​ACG​CCA​CAG​CTT​TCC

### CCK-8 detection of cell proliferation

Cells were seeded in 96-well plates at a density of 5,000 cells per well, and cell viability was determined using Cell Counting Kit-8 (CCK-8) (Sigma-Aldrich, Shanghai, China) following the manufacturer’s instructions.

### Colony formation assay

The cells were seeded in 6-well plates at a density of 500 cells per well and cultured for 10 days. After the culture was completed, the cells were stained with 0.2% crystal violet for 10 min, and then the colonies formed were counted.

### Reactive oxygen species (ROS) detection

ROS levels were detected using MitoSOX™ Red mitochondrial superoxide indicator (Invitrogen, United States of America). 1.0 × 10^5^ cells were cultured in Nunc™ glass bottom culture dishes (Invitrogen, United States) for 24 h. Before incubation, cells were washed with warm DPBS and then incubated with 2.5 µM MitoSOX™ Red. Fluorescence intensity was analyzed using a Zeiss LSM 800 confocal microscope and quantitatively measured by ImageJ software.

### Data analysis

Data were expressed as mean ± SD of three independent experiments. All experiments were repeated at least three times. The differences between groups were analyzed by Student’s t-test or one-way analysis of variance (ANOVA), and statistical analysis was performed by GraphPad Prism 9 software. Significance levels were marked as: *p < 0.05; **p < 0.01; ***p < 0.001; ****p < 0.0001.

## Results

### Identification of DEGs in the control and RPA groups

**FIGURE 1:**
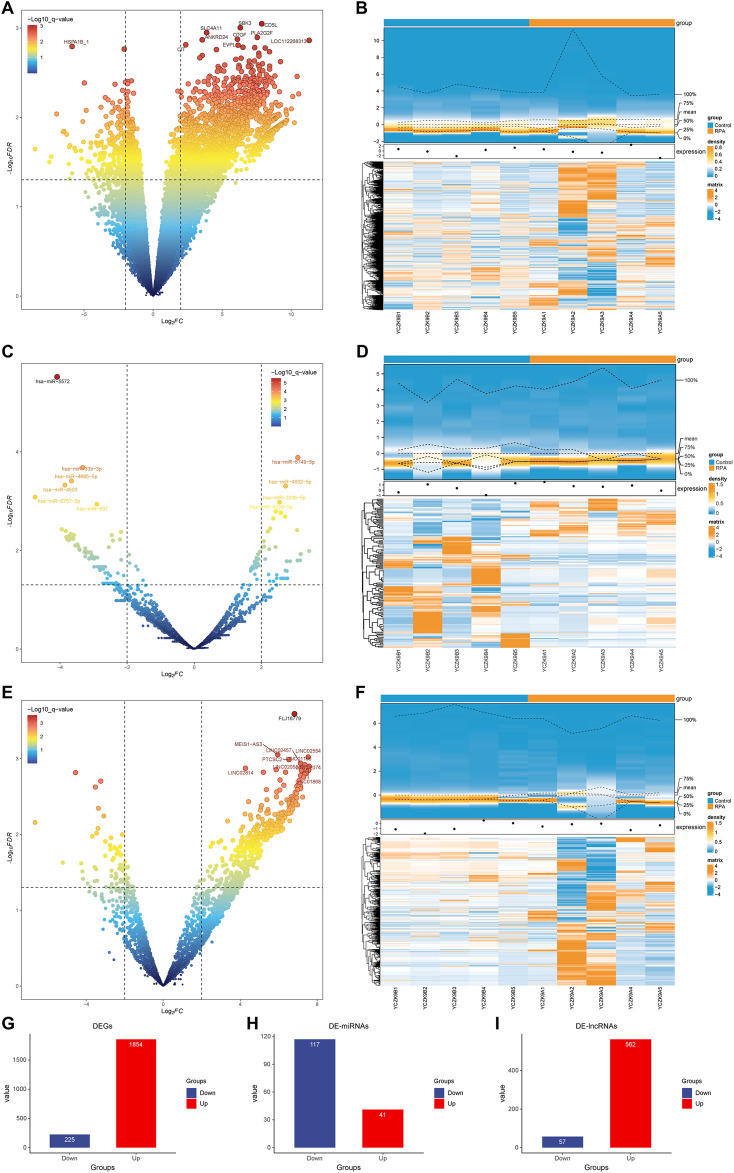
Identification of DEGs in the Control group and RPA group. **(A)** DEGs distribution volcano map, the horizontal axis represents log2FC, and the vertical axis represents -log10 (P.Value). Each dot in the figure represents a gene. The horizontal reference line represents -log10 (P.Value) = 1.3, and the vertical reference line represents log2FC = ±2. **(B)** DEGs distribution heatmap, the upper part: density distribution heatmap, showing the distribution density of gene expression in different samples, and the gene expression is normally distributed overall; the lower part: each row represents the relative expression of a gene in all samples, each column represents the relative expression of all genes in a sample, and the color of each square represents the relative expression of the gene in the sample, blue is low expression, and orange is high expression. **(C)** DE-miRNAs distribution volcano map. **(D)** DE-miRNAs distribution heatmap. **(E)** DE-lncRNAs distribution volcano map. **(F)** DE-lncRNAs distribution heatmap. **(G)** DE-mRNAs distribution bar chart statistics. **(H)** DE-miRNAs distribution bar chart statistics. **(I)** DE-lncRNAs distribution bar chart statistics.

Based on the sequencing data, the differentially expressed genes between the two groups were analyzed. For details, see Supplementary File S2. *mRNA.edgeR.csv*. Using the selection criteria of P < 0.05 and |Log2FC|≥2, a total of 2079 DEGs were identified. Compared with the Control group, 1854 DEGs were upregulated, while 225 DEGs were downregulated in the RPA group. The differential expression of genes was visualized using a volcano plots (Figure 1A) and a heatmap (Figure 1B). The miRNAs and lncRNAs in the DEGs were further identified. According to the screening conditions: |Log2FC|>2, P < 0.05, 158 DE-miRNAs were identified. Compared with the Control group, there were 41 upregulated DE-miRNAs and 117 downregulated DE-miRNAs in the RPA group (Supplementary File S3. *miRNA.edgeR.csv).* The expression of DE-miRNAs was displayed using a volcano plot (Figure 1C). Similarly, 619 DE-lncRNAs were identified based on the same standard, including 562 upregulated DE-lncRNAs and 57 downregulated DE-lncRNAs (Supplementary File S4. *lncRNA.edgeR.out.csv*). The expression of DE-lncRNAs was displayed by a volcano plot (Figure 1D) and a heat map (Figure 1E). Additionally, bar charts provided a more detailed visualization of the distribution of DE-mRNAs, DE-miRNAs, and DE-lncRNAs between the Control and RPA groups (Figures 1F–H). The significant differences in transcription levels between the groups indicated that lncRNAs, miRNAs, and mRNAs play a key role in promoting RPA.

### DEGs functional enrichment analysis

**FIGURE 2:**
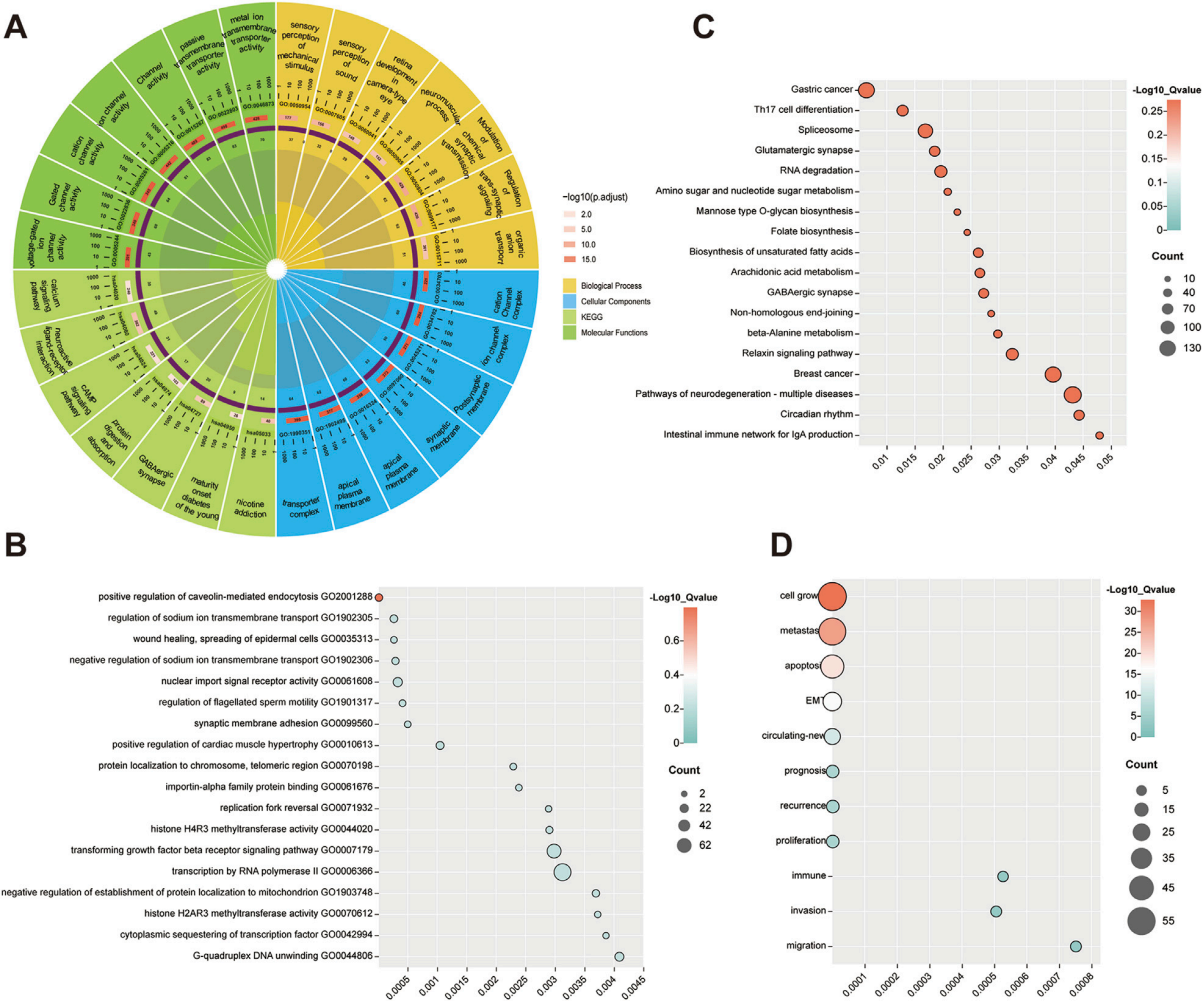
DEGs functional enrichment analysis. **(A)** Enrichment results of DEGs. The enrichment circle diagram is divided into four parts according to color: biological process, molecular function, cellular component and KEGG enrichment. The outermost ring is the enriched function, the second ring is the total number of genes contained in the function, the third ring is the number of genes enriched in the function, and the inner ring is the ratio of the number of enriched genes to the total number of genes. **(B)** GO enrichment results of DE-miRNAs. The horizontal axis is the p-value and the vertical axis is the enriched pathway. The size of the circle is related to the number of enriched factors. The more miRNAs are enriched, the larger the circle. **(C)** KEGG enrichment results of DE-miRNAs. **(D)** KEGG enrichment results of DE-lncRNAs.

DEGs were further enriched with KEGG pathways and GO functions to explore the biological significance of each gene. Using a significance threshold of p < 0.05 and count>1, the enrichment analysis identified 160 biological processes, 103 cell components, 101 cell components, and 7 KEGG pathways (Supplementary File S5. *mRNA.GO.csv* and Supplementary File S6. *mRNA.KEGG.csv)*. The top 7 GO and KEGG enrichment situations were displayed by ggplot2. These genes were significantly related to functions such as cAMP signaling pathway, protein digestion and absorption, and calcium signaling pathway (Figure 2A). For DE-miRNAs, KEGG pathways and GO enrichment analyses were conducted using the miEAA database. Under the same selection criteria, the results revealed the enrichment situation of the top 18 GO (Figure 2B) and KEGG (Figure 2C). These miRNAs were significantly related to functions such as Th17 cell differentiation, physiological rhythm, and biosynthesis of unsaturated fatty acids (Supplementary File S7. *miRNA Enrichment and Annotation.csv*). Additionally, tumor biomarker pathway enrichment analysis of DE-lncRNAs was performed using the LncSEA database, identifying 11 tumor biomarker pathways under the same selection criteria. The results indicate that these lncRNAs are significantly associated with functions such as cell apoptosis, migration, and cell growth (Figure 2D).

### Construction of cerna network in RPA

**FIGURE 3:**
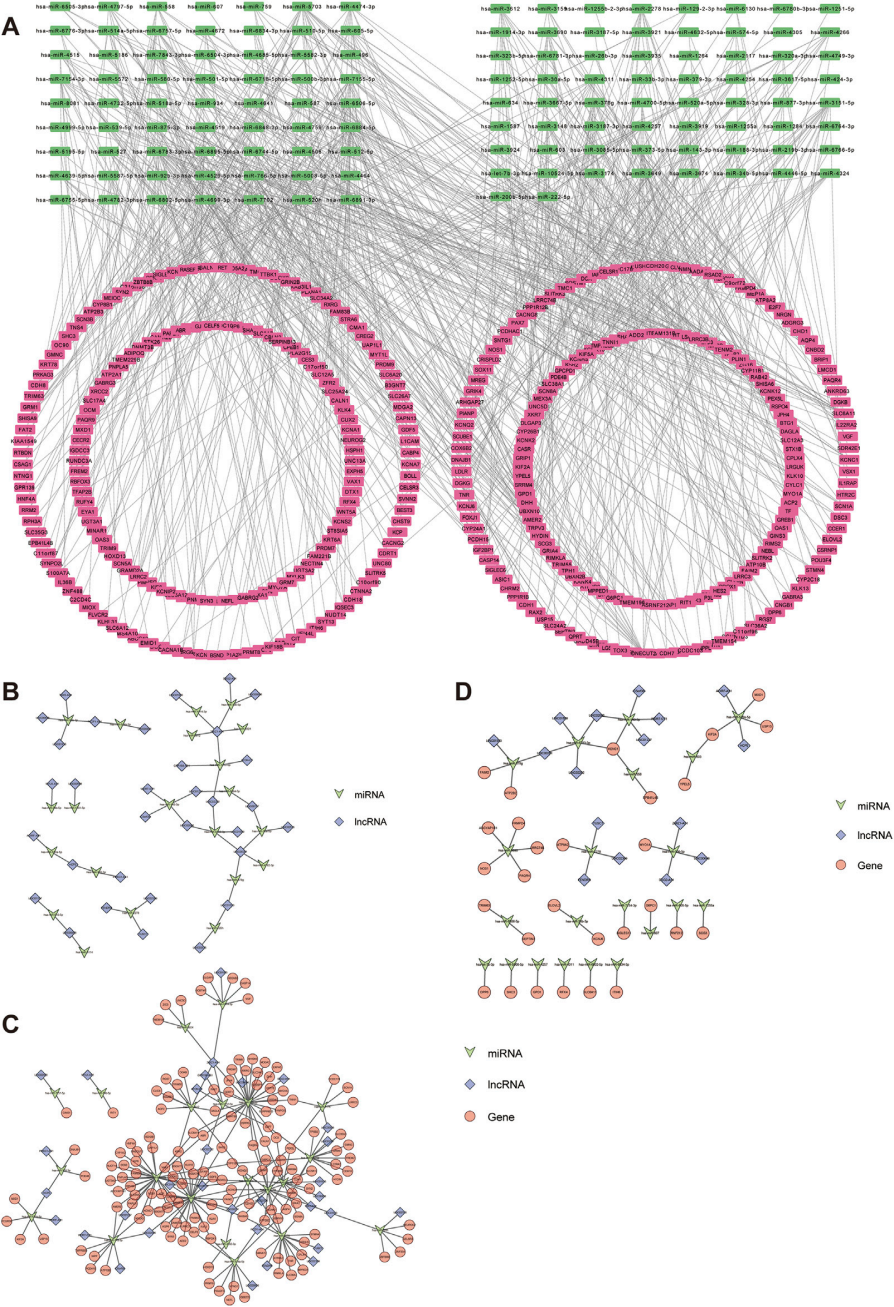
Construction of ceRNA network in RPA. **(A)** DE-miRNAs-DE-mRNAs regulatory network. **(B)** DE-miRNAs-DE-lncRNAs regulatory network. **(C)** DE-miRNAs-DE-lncRNAs-DE-mRNAs regulatory network. **(D)** Key ceRNA regulatory network.

In order to analyze the role of DE-miRNAs in the expression regulation process, the regulatory network analysis of DE-miRNAs was performed. The miRWalk database was used to predict the mRNAs that interact with differential miRNAs, and the genes predicted in both the miRWalk and miRDB databases were selected as the predicted mRNAs. A total of 7,912 mRNAs were predicted (Supplementary File S8. *miRNA-mRNA.xls*). By intersecting predicted mRNAs with DE-mRNAs, 387 mRNAs, 129 miRNAs, and 743 miRNAs-mRNAs interactions were identified. Finally, the Cytoscape software was used to construct the upstream regulatory network of DE-mRNAs and intersection miRNAs (Figure 3A). The tarbase database is a database that specifically collects miRNAs-RNAs targeting relationships supported by experimental evidence. This study searched lncRNAs regulated by differential miRNAs through the tarbase database and the ENCORI database, and obtained 48 miRNA-lncRNA regulatory relationships after taking the intersection with the differential lncRNAs (Supplementary File S9. *miRNA-lncRNA. xls*). Then, the Cytoscape software was used to construct the upstream regulatory network for the differential miRNAs and the intersection lncRNAs (Figure 3B).

The results of the above two regulatory networks were integrated, and the factors that could construct the DE-miRNAs-DE-lncRNAs-DE-mRNAs network were selected to draw the ceRNA network regulation diagram (Figure 3C). In Figure 3A, miRNAs and mRNAs in the miRNAs-mRNAs network were further selected, and the correlation between the two was analyzed by spearman. The correlation coefficients with an absolute value greater than 0.6 and p ≤ 0.05 were selected as key regulatory networks, and 29 mRNAs, 21 miRNAs, 16 lncRNAs, and 32 miRNAs-mRNAs regulatory relationships were identified. The above relationships were combined to construct the ceRNA network (Figure 3D).

### PPI analysis and construction of related networks

**FIGURE 4:**
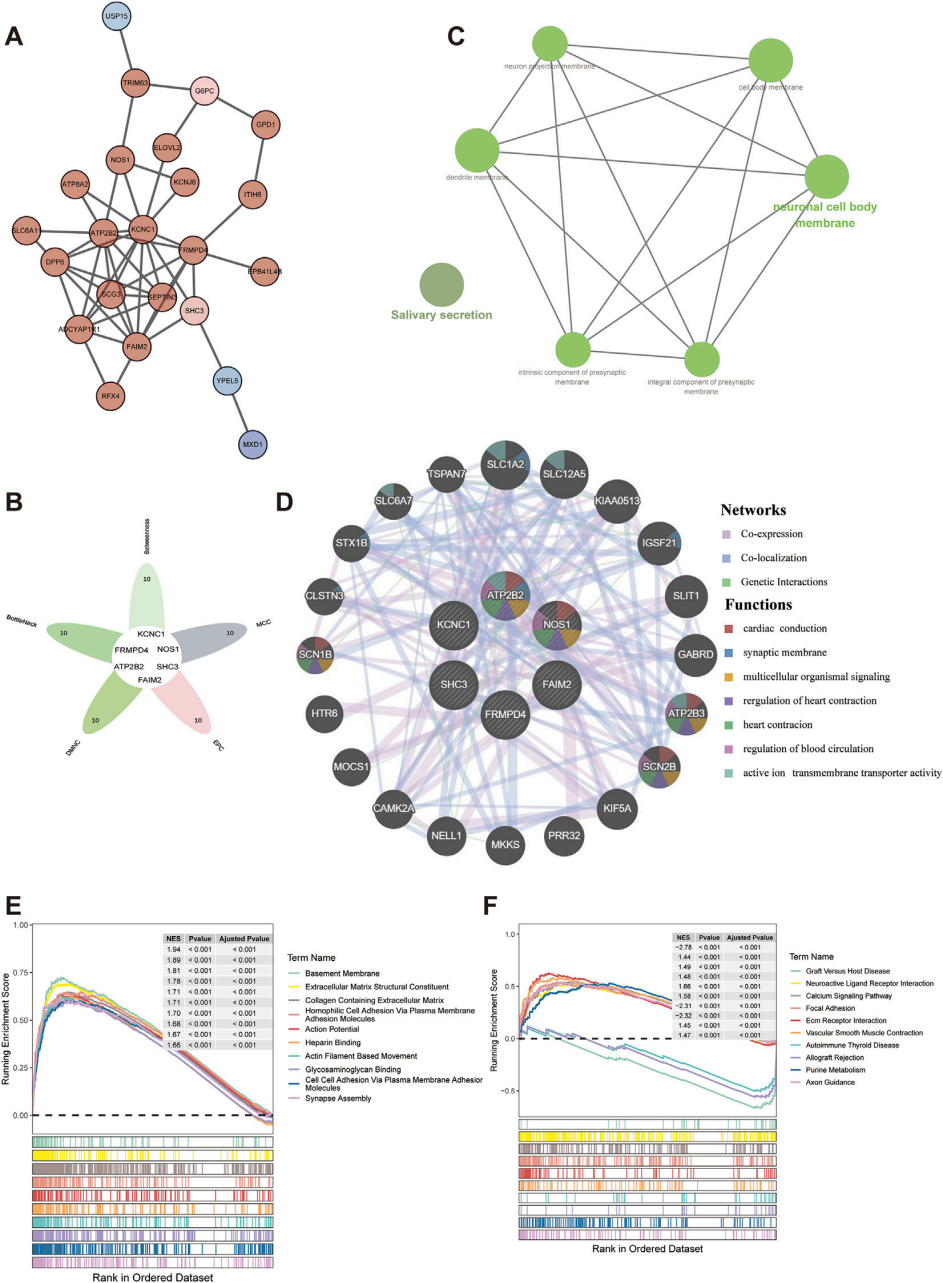
PPI analysis and related network construction. **(A)** PPI network of 29 DEGs in the key regulatory network. **(B)** Venn diagram showing the screened key genes. **(C)** ClueGo pathway enrichment analysis diagram of key genes. **(D)** GeneMANIA enrichment results of key genes. **(E)** GO enrichment analysis results of key gene ATP2B2. **(F)** KEGG enrichment analysis results of key gene ATP2B2.

A PPI network was constructed for the 29 DEGs in the key regulatory network to analyze the interactions between genes, and the PPI network was further beautified by cytoscape software (Figure 4A). The 29 genes in the key regulatory network were screened using the algorithm of Cytoscape software. The top 10 genes were obtained for each algorithm, and the overlapping genes in the algorithm were recorded as key genes. After drawing the Venn diagram, 6 key genes were obtained: KCNC1, FRMPD4, ATP2B2, NOS1, SHC3, and FAIM2 (Figure 4B). ClueGO functional enrichment analysis was performed on the key genes, and the results showed that these genes were mainly related to functions such as synapses and cell membranes (Figure 4C) (Supplementary File S10. *ClueGO.ResultTable.xls*).

Next, a functional analysis was conducted to assess whether these key genes could regulate other genes. The GeneMANIA database was used to identify 20 interacting genes and the top 5 most significantly associated pathways for each key gene. The six key genes were mainly significantly related to cardiac conduction and cardiac contraction regulation (Figure 4D).

In order to further explore some related signaling pathways and potential biological mechanisms of key genes. Single gene GSEA enrichment analysis was performed on key genes, with thresholds set to |NES|>1, NOM P < 0.05, and q < 0.25. ATP2B2 was used for single gene GSEA enrichment analysis and 1005 GO enrichments and 27 KEGG pathways were obtained (Supplementary File S11. *gsea.ATP2B2.go.txt* and Supplementary File S12. *gsea.ATP2B2.kegg.txt*). The top 10 significant GO enrichments (Figure 4E) and KEGG (Figure 4F) pathways were selected for mapping. The results showed that ATP2B2 was mainly related to calcium signaling pathways, focal adhesions, vascular smooth muscle contraction and other functions. The enrichment results of the remaining genes are shown in Supplementary Figure S1.

### Analysis of key gene functions

**FIGURE 5:**
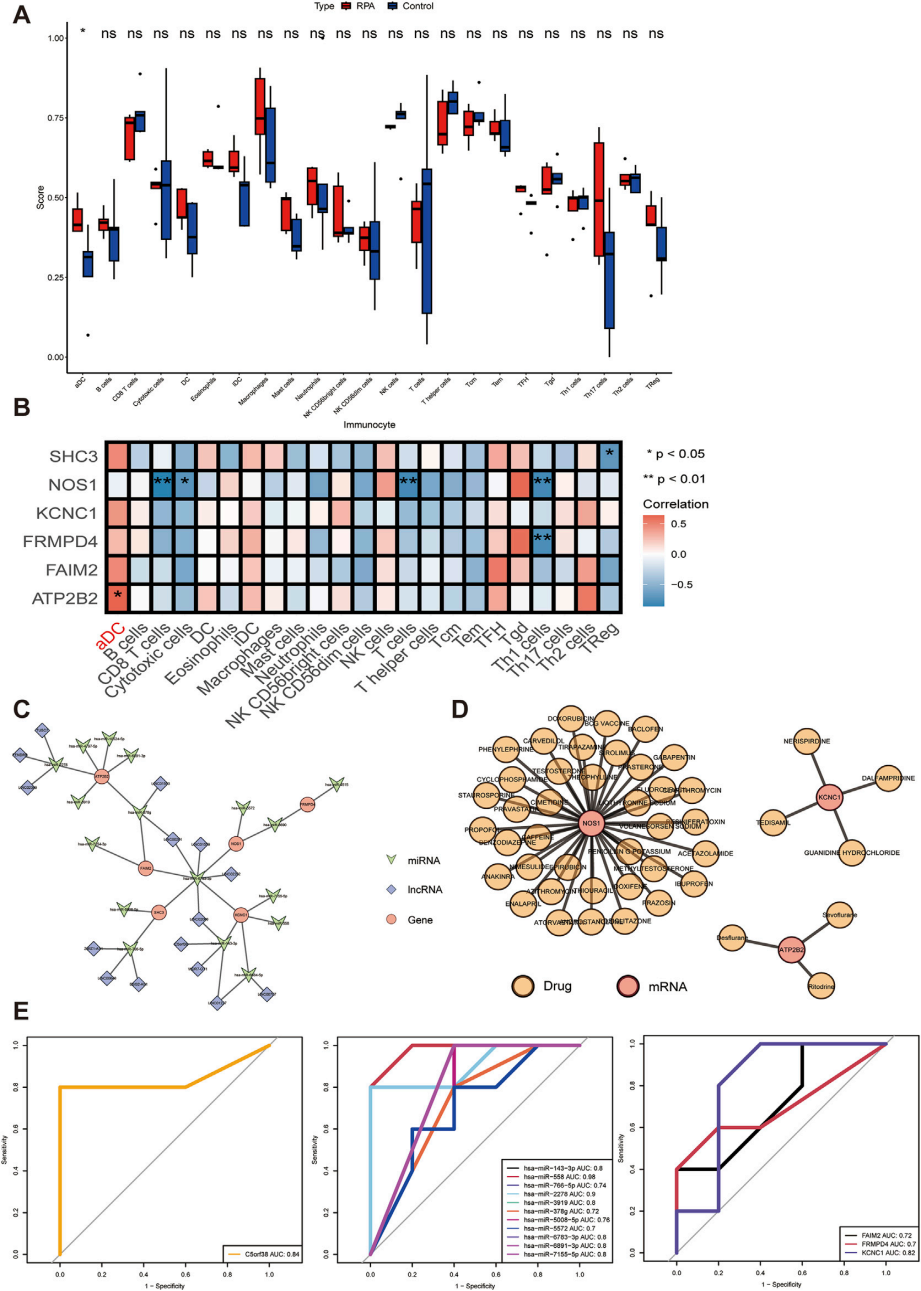
Analysis of key gene functions. **(A)** Box plot of immune cell proportions between groups. **(B)** Heatmap of correlation between key genes and immune cells. **(C)** Upstream regulatory network of key genes. **(D)** Drug-gene interaction network. **(E)** ROC curves of key genes, miRNAs, and lncRNAs.

Microenvironment cells are crucial components of tissues, and increasing evidence has clarified their clinical pathological significance in predicting prognosis and treatment effects (Tong et al., 2023). The ssGSEA method was applied to estimate the activity of immune cells, immune functions, and immune pathways in each sample. Based on the activity levels, samples were categorized into groups. By analyzing these immune-related gene sets, the immune activity of each sample can be obtained. Based on 24 immune cell datasets, the ssGSEA algorithm was used to calculate the abundance percentage of infiltrating immune cells in each sample (Supplementary File S13. *immune_ssgseaOut.csv*). A comparative analysis between the two groups showed aDC immune cells exhibited significant differences, suggesting that aDC immune cells may play a key role in RPA (Figure 5A). Spearman correlation was further used to analyze the correlation between key genes and immune cells. The correlation coefficient is shown in Supplementary File S14. *gene.ssGSEA.correlation.csv*. The analysis found that the key gene ATP2B2 was significantly positively correlated with the proportion of aDC immune cells (Figure 5B).

A key gene subnetwork was extracted from the overall ceRNA regulatory network by isolating the key genes and their related nodes (including lncRNAs and miRNAs). The resulting subnetwork involved 6 mRNAs, 17 miRNAs, and 1 lncRNA (Figure 5C).

In order to explore potential therapeutic drugs for diseases related to the four key genes, compound screening was performed for the proteins encoded by these key genes. The TissueNexus database was used to predict drugs acting on key genes and a gene-drug interaction network diagram was constructed (Figure 5D). A total of 45 targeted drugs were identified to have therapeutic effects on the three key genes. The KCNC1 gene has 4 target drugs, the NOS1 gene has 38 target drugs, and the ATP2B2 gene has 3 target drugs. The remaining genes have no corresponding target drugs predicted. Detailed target drug information is shown in Supplementary File S15. *TissueNexus.xls*. These drugs may be beneficial for future patient treatment strategies.

In order to study the ability of key genes, key miRNAs, and key lncRNAs to distinguish between slower RPA samples and faster RPA samples, this study used the pROC R package (version 1.18.0) in the self-test data set to draw the ROC curves of the two groups of samples. The area under the ROC curve represents the AUC value. The larger the AUC value, the more accurate the prediction. Generally, an AUC value greater than 0.7 indicates a strong ability to distinguish. In the ceRNA network of key genes, the AUC values of 11 miRNAs were all greater than 0.7, the AUC value of 1 lncRNA was greater than 0.7, and the AUC values of 3 mRNAs were all greater than 0.7 (Figure 5E).

### Clinical sample validation of key gene functions

**FIGURE 6:**
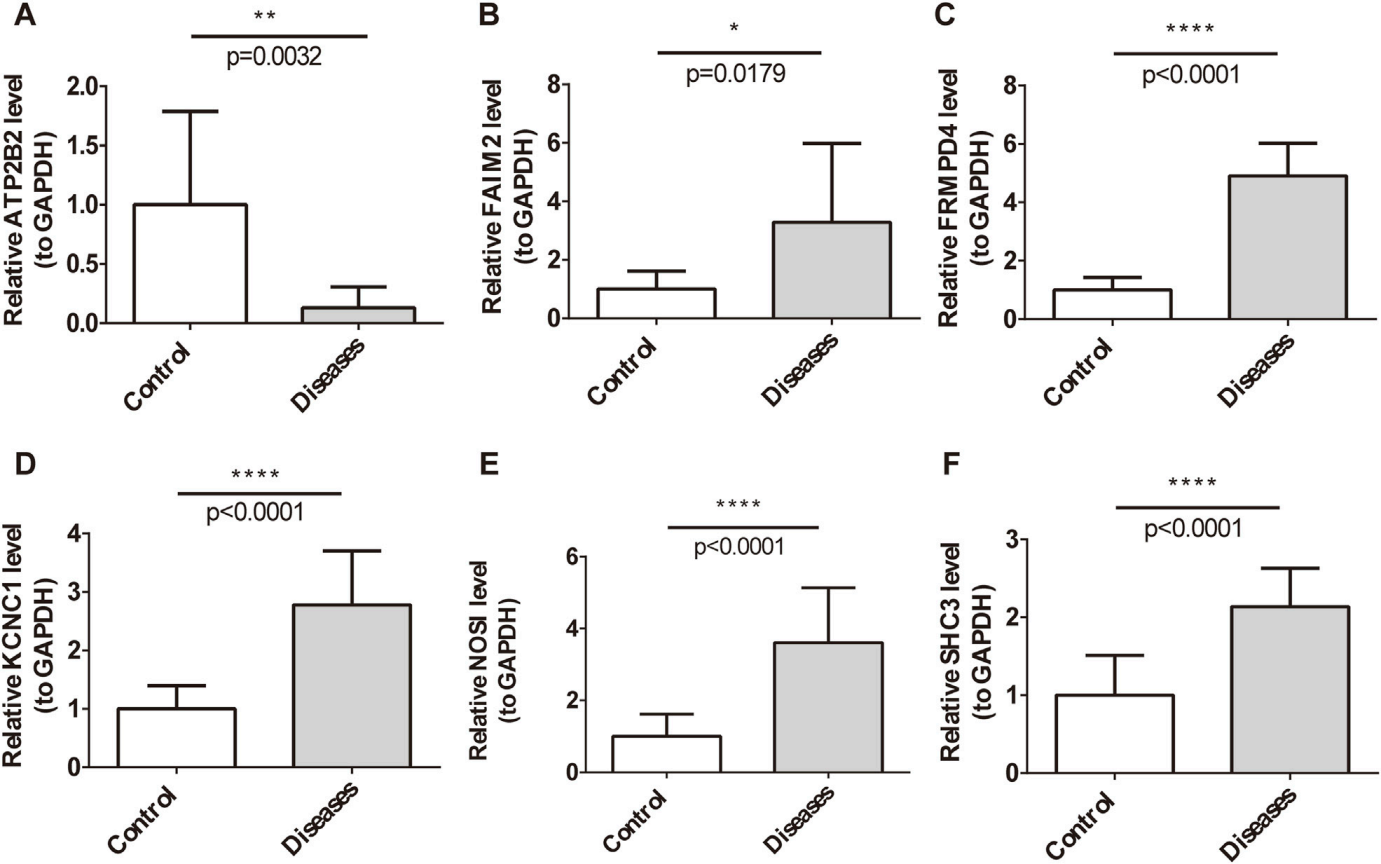
Clinical sample validation of key gene functions. **(A–F)** Bar graphs showing the mRNA expression levels of six key genes in RPA and Control groups.

In order to further verify the biological function of key genes, 10 tissue samples of RPA patients and healthy controls were obtained after PTA surgery. The expression of key genes, miRNAs, and lncRNAs was detected by qRT-PCR. The results showed that compared with the Control group, except for ATP2B2, the mRNA expression levels of KCNC1, FRMPD4, NOS1, SHC3, and FAIM2 in the RPA group were significantly increased, indicating that the disease progression of restenosis may be promoted (Figures 6A–F).

## Discussion

Restenosis of peripheral arteries (RPA) is a common complication after peripheral arterial interventional therapy, which seriously affects the prognosis of patients (Jia et al., 2022; Zhou et al., 2021). Despite recent advancements in the prevention and treatment of RPA, its specific molecular mechanism has not been fully elucidated and there is a lack of effective prognostic biomarkers. Since the ceRNA hypothesis was proposed, researchers have become increasingly interested in the ceRNA network, in which lncRNAs may affect the transcription and expression of mRNAs by interacting with miRNAs (Tong et al., 2023) ceRNA is an important mechanism by which lncRNAs regulate gene expression and may have a huge impact on peripheral arterial disease. It has been widely reported that the disorder of the ceRNA network is closely related to the progression of arterial disease (Zhong et al., 2021). Therefore, the ceRNA network may serve as a novel tool to understand the potential mechanism of RPA and to identify potential therapeutic targets. This study constructed a ceRNA network based on an RNA expression dataset by identifying genes that were significantly differentially expressed in healthy controls and samples with RPA. By constructing a ceRNA regulatory network, key molecules closely related to the occurrence and development of RPA were screened out, providing new ideas for exploring the molecular mechanism of RPA and finding potential therapeutic targets.

The rapid development of bioinformatics methods has provided methodological support for exploring high-throughput sequencing data (Huang et al., 2024). The differential RNA expression observed between samples with fast and slow RPA suggests that differentially expressed genes may play a key role in the progression of restenosis. This study used whole transcriptome data from 5 healthy control tissue samples and 5 tissue samples from patients with RPA to study the potential molecular regulatory mechanisms in RPA. 2079 DEGs, 158 DE-miRNAs, and 619 DE-lncRNAs were screened in the RPA group and the Control group. Enrichment analysis was then conducted on these DEGs and DEG-ncRNAs to obtain the relevant functional pathways. A differential gene regulatory network was subsequently constructed, the candidate differential genes were screened in the PPI network using Cytoscape software, and 6 key genes were obtained: KCNC1, FRMPD4, ATP2B2, NOS1, SHC3, and FAIM2. The GSEA enrichment analysis was used to identify the function of each key gene. From the analysis of immune cell infiltration, it was found that aDC immune cells were significantly different in the two groups of samples, and there was a significant positive correlation between the key gene ATP2B2 and the proportion of aDC immune cells, reflecting that immune cells play a certain role in RPA. With the help of DE-ncRNA, the upstream regulatory network of key genes was constructed in combination with database prediction. Finally, the small molecule drugs for key genes were predicted in the TissueNexus database, and 45 target drugs were found to have potential effects on the treatment of RPA.

This study identified six key genes: KCNC1, FRMPD4, ATP2B2, NOS1, SHC3, and FAIM2, explored the potential molecular regulatory mechanisms in RPA, and analyzed the biological pathways and regulatory networks involved in the key genes, providing a theoretical basis for the diagnosis and treatment of RPA. The clinical diagnostic analysis of key genes found that the AUC value of the ROC curve was greater than 0.8, indicating that it had a good predictive value for the occurrence of restenosis. The mRNA levels of key genes in the Control group and RPA group were detected by qRT-PCR. The results showed that the mRNA expression levels of UPCAT1, PLA2G1B, and SMMPD4 were significantly increased in the fast group, indicating that the disease progression of restenosis may be promoted. These findings indicate that the six key genes may serve as clinical prognostic factors for RPA and represent potential therapeutic targets for its treatment.

## Data Availability

The original contributions presented in the study are included in the article/Supplementary Material, further inquiries can be directed to the corresponding author.
