# Supplementary figures and images for: Potential prognostic biomarkers in restenosis of peripheral arteries identified through comprehensive ceRNA network analysis
Source: Front Genet. 2025 Dec 1;16:1597644. doi: 10.3389/fgene.2025.1597644 (PMC12702502; doi:10.3389/fgene.2025.1597644)

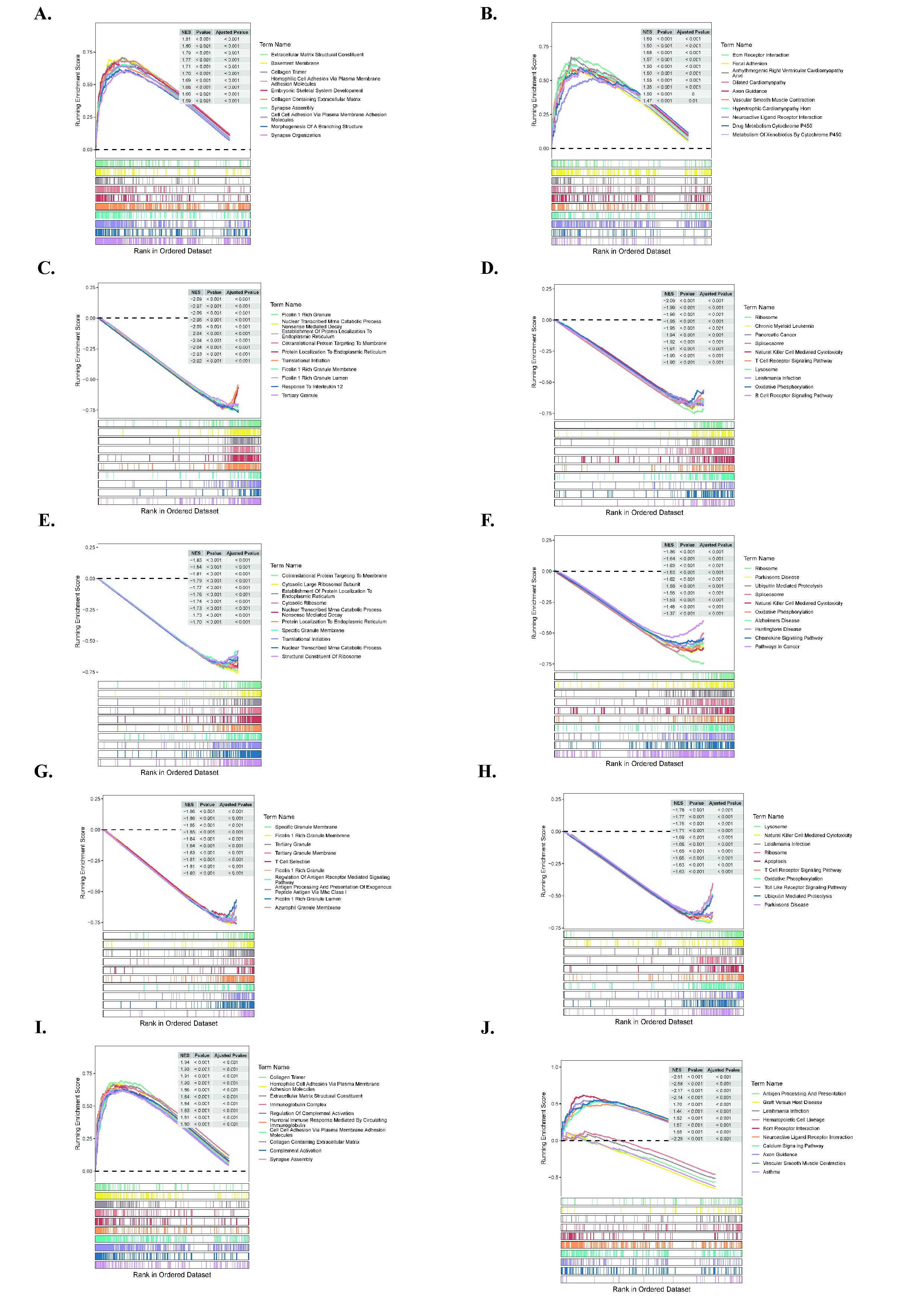

Supplement: Supplementary file 5 [file Image1.jpeg]
